# *Déjà-lu:* When Orthographic Representations are Generated in the Absence of Orthography

**DOI:** 10.5334/joc.250

**Published:** 2023-01-12

**Authors:** Mina Jevtović, Alexia Antzaka, Clara D. Martin

**Affiliations:** 1 Basque Center on Cognition, Brain and Language (BCBL), Donostia-San Sebastián, Spain; 2 University of the Basque Country (UPV/EHU), Leioa, Spain; 3 Universidad Internacional de La Rioja, Logroño, Spain; 4 Basque Foundation for Science (Ikerbasque), Bilbao, Spain

**Keywords:** word learning, orthographic representations, spelling, reading, phonology

## Abstract

When acquiring novel spoken words, English-speaking children generate preliminary orthographic representations even before seeing the words’ spellings (Wegener et al., 2018). Interestingly, these *orthographic skeletons* are generated even when novel words’ spellings are uncertain, at least in transparent languages like Spanish (Jevtović et al., 2022). Here we investigate whether this process depends on the orthographic rules of the language, and specifically, whether orthographic skeletons for words with uncertain spellings are generated even when the probability of generating an incorrect representation is high. Forty-six French adults first acquired novel words via aural instruction and were then presented with words’ spellings in a self-paced reading task. Importantly, novel words were presented in their unique (consistent words) or one of their two possible spellings (preferred and unpreferred inconsistent words). A significant reading advantage observed for aurally acquired words indicates that participants indeed generated orthographic representations before encountering novel words’ spellings. However, while no differences in reading times were found for aurally acquired words with unique and those presented in their preferred spellings, unpreferred spellings yielded significantly longer reading times. This shows that orthographic skeletons for words with multiple spellings were generated even in a language in which the risk of generating an incorrect representation is high. This finding raises a possibility that generating orthographic skeletons during spoken word learning may be beneficial. In line with this conclusion is the finding showing that – in interaction with good phonological short-term memory capacity – generating orthographic skeletons is linked to better word recall.

## 1. Introduction

The present study explores the influence of orthographic knowledge on auditory word learning. By manipulating the spelling of aurally acquired words, we test whether adult skilled readers generate orthographic representations for novel spoken words even in the absence of orthography. Contrary to learning novel words during early childhood, acquiring novel vocabulary as an adult is usually accompanied with orthographic information. Much of the previous literature comparing word learning with and without orthographic labels, has shown that when presented along with their phonological forms, novel words’ spellings facilitate the learning process (Ehri & Wilce, 1979; Ricketts, Bishop, & Nation, 2009; Rosenthal & Ehri, 2008). Positive effects of orthography on novel word learning have been observed in both children (Ricketts, Bishop, & Nation, 2009; Rosenthal & Ehri, 2008) and adults (Nelson, Balass, & Perfetti, 2005). Interestingly, this *orthographic facilitation* in vocabulary learning seems to be robust since it emerges even in populations with reading disorders (e.g., developmental dyslexia; Baron et al., 2018). Previous research thus shows that directly and explicitly linking orthography to novel phonological word forms serves as an additional memory trace that facilitates the acquisition of novel words.

Recently, there has been some interest in the indirect effects of orthography on spoken word learning. Namely, several studies have shown that orthographic knowledge affects spoken word learning even before the first visual exposure with words’ actual spellings. By converting sounds-to-letters, a mechanism similar to the one employed when decoding novel written words (see Share, 1995) is able to generate *orthographic skeletons* (i.e., preliminary orthographic representations) solely from aural exposure to novel word forms (Beyersmann et al., 2021; Jevtović, Antzaka, Martin, 2022; Wegener et al. 2018; Wegener, Wang, Nation, & Castles, 2020, but see also Johnston, McKague, & Pratt, 2004).

This idea, first proposed by Stuart and Coltheart (1988) and known as the *Orthographic Skeleton Hypothesis*, was directly tested by Wegener and colleagues (2018; see also Johnston, McKague, & Pratt, 2004; Wegener et al., 2020). In their eye tracking study, the authors trained a group of fourth-grade children on meanings and pronunciations of novel words without exposing them to words’ spellings. When these aurally acquired words were later presented in a sentence reading task, printed forms of words with predictable spellings (e.g., /neʃ/ spelled as <nesh>) were read faster than words with unpredictable spellings (e.g., /kɔɪb/ written as <koyb> rather than <coib>). The same facilitation for predictable spellings did not occur in the set of untrained words thereby yielding a significant interaction between training and spelling predictability. The authors concluded that children had generated orthographic skeletons solely based on novel trained words’ phonology. They argued that orthographic skeletons children had generated for words with predictable spellings were confirmed in the subsequent reading task thus leading to their faster reading times and significant training facilitation. In contrast, orthographic skeletons children had generated for words with unpredictable spellings did not match those seen in the reading task as indicated by the absence of training facilitation. Nonetheless, it is possible that longer reading times observed for the unpredictable spellings were not due to children forming expectations that did not match the real spelling, but rather the fact that they did not form any expectations at all for items with unpredictable spellings. In other words, whether orthographic skeletons are generated for all words acquired through aural instruction or only for words with highly predictable spellings remained an open question.

To adjudicate between these two possibilities Jevtović et al. (2022) created a similar experiment in Spanish which allowed them to control for the number of possible spellings by creating novel words with a unique (hereafter *consistent words*) or two possible spellings (hereafter *inconsistent words*). In their study, participants first acquired words through aural training and were then presented with their spellings in a short self-paced reading task. Importantly, inconsistent words were presented in each participant’s preferred (i.e., likely to be predicted) or unpreferred (i.e., unlikely to be predicted) spellings, this way yielding three groups of novel words (i.e., consistent, inconsistent preferred, and inconsistent unpreferred). As significantly longer reading times were observed only for previously acquired words shown in their unpreferred spellings, and that no differences were found in reading aurally acquired words with a unique and those shown in their preferred spellings, the authors concluded that participants had generated orthographic skeletons for all aurally acquired words (i.e., both consistent and inconsistent). That is, participants generated orthographic skeletons both for words whose spellings can be predicted with 100% certainty, like /dalu/ which can only be spelled as <dalu> in Spanish, but also for words for which there is a 50% chance of correctly predicting the actual spelling, like /bamu/ which can be spelled <bamu> or <vamu>).

Despite showing that orthographic skeletons are generated even when novel words’ spellings are uncertain, the results from Jevtović et al. (2022) are limited to skilled readers of transparent orthographies such as Spanish. In transparent orthographies, where most sounds map onto only one grapheme therefore making them highly consistent for spelling, the probability of generating an incorrect spelling expectation (i.e., an incorrect orthographic skeleton) is very low. It could be the case that generating orthographic skeletons even for words with more than one possible spelling, and then storing these representations in memory, is specific to readers of transparent orthographies (i.e., orthographies with mostly consistent sound-to-letter mappings). Speakers of languages with a high level of sound-to-spelling inconsistency, such as English or French, might not engage in the process of generating orthographic skeletons when multiple spellings are possible. Given the high number of multiple sound-to-spelling mappings, the probability of generating an incorrect skeleton in an opaque language is high. Generating, and storing in memory incorrect orthographic skeletons which then need to be updated when the actual (i.e., correct) spellings are encountered (Wegener et al., 2020), may not be beneficial for the cognitive system. Speakers of opaque languages may therefore refrain from generating skeletons, at least for inconsistent words. It could be the case that during spoken word acquisition orthography is accessed and stored in memory only if one spelling is possible (i.e., for consistent words). Alternatively, one of the multiple inconsistent word’s spellings could be preferred and thus associated with the auditory word form in the memory. Whether orthographic skeletons for words with more than one spelling are generated and stored in memory even when most words could be spelled in many different ways (opaque language) will be tested in the present study.

Compared to Spanish, French orthography is highly inconsistent for spelling (sound-to-spelling inconsistency; see Ziegler, Jacobs, & Stone, 1996). Due to many sounds having more than one possible grapheme representation (e.g., the sound /o/ has at least three grapheme representations <o>, <au> and <eau>) predicting the correct spelling of a newly acquired French word comes with higher degree of uncertainty, and consequently risk of error, as compared to Spanish. At the same time, French is relatively consistent for reading (spelling-to-sound consistency). In fact, in the majority of cases (except for irregular words which represent exceptions), relying exclusively on grapheme-to-phoneme conversion rules when decoding a novel word in French leads to a correct pronunciation, thereby making French more similar to Spanish when it comes to reading. Given its one-direction inconsistency, French is a perfect candidate for testing whether orthographic skeletons are generated when there is uncertainty regarding a novel word’s spelling even in a language with higher level of uncertainty stemming from sound-to-spelling inconsistency. At the same time, testing French speakers allows us to control for the inconsistency present in spelling-to-sound mappings.^1^ Since this is not possible in English, a two-way inconsistent language used in the original study by Wegener and colleagues (2018), the present study aims to confirm and expand on the orthographic skeleton hypothesis in another opaque language.

### 1.1. The present study

The present study further investigates the orthographic skeleton hypothesis by testing whether orthographic skeletons for words with more than one legal spelling are generated even in a language with high degree of sound-to-letter inconsistency (i.e., French). To that end, we employed the same design as the one used by Jevtović et al. (2022) and created three groups of novel French words. Words from the first group were created using sounds with a unique grapheme representation, or at least a highly dominant one (e.g., sounds /b/ and /d/ can only be written as <b> and <d> respectively in French; sound /i/ can be spelled <i> and <y>, however <i> represents the most common spelling). Consequently, those words had one dominant spelling (e.g., /bemanə/ spelled as <bémane>). By contrast, initial sounds of all inconsistent words had two possible grapheme representations (e.g., sound /ʒ/ can be spelled either with the letter <g> or the letter <j>) giving this way items with two equally plausible spellings (e.g., /ʒebinə/ can be written as <gébine> or <jébine>). Half of the inconsistent words were presented in each participant’s preferred spelling (inconsistent preferred words) while the other half was presented in their unpreferred spellings (inconsistent unpreferred words). Individual spelling preferences were obtained on a separate experimental session completed two weeks before the aural training took place (i.e., Session 1). Two weeks later, during Session 2, participants acquired the words via aural training and were then exposed to their spellings in a self-paced reading task. Apart from reading previously (aurally) acquired words, participants were also presented with the three groups of untrained words (consistent, inconsistent preferred, and inconsistent unpreferred).

Following the same reasoning as Jevtović et al. (2022), we hypothesised that if orthographic skeletons are generated even for words with more than one possible spelling, reading times for previously acquired consistent and inconsistent preferred words should not differ. Trained words visually presented in their inconsistent unpreferred spellings should however yield longer reading times as there will be a mismatch between the orthographic skeleton participants generated and the spellings they were presented with. If, however, orthographic skeletons are generated only when there is low risk of generating an incorrect expectation (i.e., only in fairly transparent languages), aurally trained consistent French words should be read faster than the inconsistent ones altogether. Importantly, in both cases, there should not be any differences in reading words from the untrained set of words, thus yielding a significant interaction between training and word group. If orthographic skeletons are generated regardless of the number of possible spellings, this interaction should, in line with Wegener et al. (2018), be driven by a facilitation present when reading words in line with participants’ spelling expectations (i.e., consistent and preferred). Alternatively, the same interaction could also stem from longer reading times (i.e., inhibitory effect) present only for inconsistent unpreferred spellings, as observed in Jevtović et al. (2022). If on the other hand orthographic skeletons are generated only for words with one dominant spelling, the interaction between training and word group should be driven either by facilitation present only for consistent trained words, or by longer reading times present for all previously acquired inconsistent words.

As a secondary and exploratory goal, we set out to investigate the link between generating orthographic skeletons and novel word recall. Precisely, we tested whether participants with higher tendency to generate orthographic skeletons also remember better the newly acquired words at the end of the experiment. In addition, we wanted to explore whether individual tendency to generate orthographic skeletons is related to a construct already shown to positively correlate with spoken word learning, that is, *phonological short-term memory (PSTM)*. Better PSTM capacity, which represents a person’s ability to temporarily store phonological information, has been linked to better spoken word learning in both children (Gathercole, 2006; Gathercole & Baddeley, 1989) and adults (Baddeley, Papagno, & Vallar, 1988). However, the positive link between the two tends to decline as children get older and gain access to more advanced word learning mechanisms (e.g., comparison with similarly sounding familiar words; see Gathercole, 2006). Since this decline coincides with the official onset of reading acquisition, we hypothesized that the link between PSTM and word learning may be partly modulated by an individual’s tendency to generate orthographic skeletons, at least in adult skilled readers. As orthographic knowledge increases, and perhaps the ability to generate orthographic skeletons, PSTM may be less involved in word learning. The two in combination may thus be important predictors of spoken word learning and there should be a significant interaction between the two in predicting word recall. Participants with higher PSTM capacity scores and at the same time stronger tendency to generate orthographic skeletons would be better at recalling the words at the end of experiment. Alternatively, the tendency to generate orthographic skeletons and the PSTM capacity may be completely independent predictors of word learning outcomes, in which case there should be no interaction between the two in predicting the learning outcome. This would suggest that they represent compensatory mechanisms available to us when learning novel spoken words (i.e., one can be used when the other is not available).

## 2. Methods

### 2.1. Participants

A total of 55 participants completed two experimental sessions. However, due to technical problems, data from five participants failed to be transferred to the online testing server. An additional four participants were removed due to low accuracy in the learning phase (<70%).^2^ Data reported here come from 46 participants (39 female; M*age* = 22.8, SD = 2.63) who completed both sessions with a 10 to 14 days delay between them. All participants were recruited via announcements posted on social media (e.g., student Facebook groups and Twitter) and were given a detailed description of the study before confirming their participation. All participants had French as their first and dominant language and all completed a questionnaire on their language skills and habits before starting Session 1 (see Table 1 for more details on participants’ self-reported measures of proficiency). The study was approved by the BCBL Ethics Review Board (approval number 060420 MK) and complied with the guidelines of the Helsinki Declaration. All participants gave their written consent at the beginning of each experimental session. They received 20 euros for their participation.

**Table 1:** Summary of Subjective (self-rated) Measures of Participants’ Proficiency in French.


	MEAN	SD	RANGE

**Age of Acquisition**	0.00	0.00	0–0

**Self-rated proficiency**(0–10)*			

*Speaking*	9.33	0.790	7–10

*Understanding*	9.61	0.576	8–10

*Writing*	8.93	0.998	7–10

*Reading*	9.50	0.753	8–10


*Note*: Some participants had low to intermediate knowledge of English. However, none of them was fully proficient in English or any other language.

### 2.2. Stimuli

#### 2.2.1. Novel words

Two sets of 24 six phoneme CVCVCV disyllabic novel French words were created (set A and B). Each set contained 8 consistent and 16 inconsistent words (see Table 2). All consistent words were made of sounds with one dominant grapheme representation in French (hereafter consistent sounds) and therefore they all had one dominant spelling (e.g., word /danyvə/ as <danuve>). Initial sounds of all inconsistent words had two possible grapheme representations while the remaining four sounds were consistent, giving this way words with two possible spellings. More specifically, all inconsistent words started with one of the following four sounds: /ʒ/ which can be written as either <g> or <j>, /k/ which followed by /i/ or /e/ maps onto either <qu> or <k>, /f/ which can be represented with a letter <f> or letters <ph>, and /s/, which followed by /i/ or /e/ can be written with either <c> or <s>.

**Table 2:** Two Sets of Novel Words Used in the Experiment.


SET	CONSISTENT	INCONSISTENT PREFERRED	INCONSISTENT UNPREFERRED

**A**	/bemanə/	/ʒinavə/	/ʒitymə/

	/danyvə/	/ʒebinə/	/ʒevabə/

	/tunavə/	/sedunə/	/semivə/

	/mabynə/	/simybə/	/sibavə/

	/nypinə/	/fapyvə/	/fanynə/

	/vetagə/	/fedinə/	/fenɔgə/

	/pivadə/	/kityvə/	/kidunə/

	/lybavə/	/kemagə/	/kepydə/

**B**	/badivə/	/ʒimunə/	/ʒitɔgə/

	/devabə/	/ʒedavə/	/ʒenyvə/

	/mevinə/	/sitavə/	/sidynə/

	/nemunə/	/sepidə/	/sebavə/

	/tabynə/	/fabɔgə/	/fapunə/

	/pinagə/	/fenybə/	/febadə/

	/lapyvə/	/kipynə/	/kimavə/

	/vinyvə/	/kenivə/	/kepanə/


*Note*: Words from the inconsistent preferred group were later shown in each participant’s preferred spelling while words from the inconsistent unpreferred group were presented in participants’ unpreferred spelling.

Ten pilot participants who did not participate in the main study completed a pretest spelling task and confirmed the consistent versus inconsistent spelling manipulation. They all spelled the consistent words in the same way (e.g., /bemanə/ spelled as <bémane>), and for the inconsistent words they provided one of the two dominant spellings (e.g., /ʒebinə/ would be spelled as either <gébine> or <jébine>).

Consistent words from the two sets were matched for the number of orthographic neighbors (Set A: M = .250, SD = .707 and Set B: M = .375, SD = .744, *t*(14) = –.344, *p* = .736) and neither of the possible spellings of inconsistent items had more than two orthographic neighbors. All words were recorded by a female native French speaker in a sound attenuated cabin using Marantz PMD661.

#### 2.2.2. Novel objects

**Figure 1:**
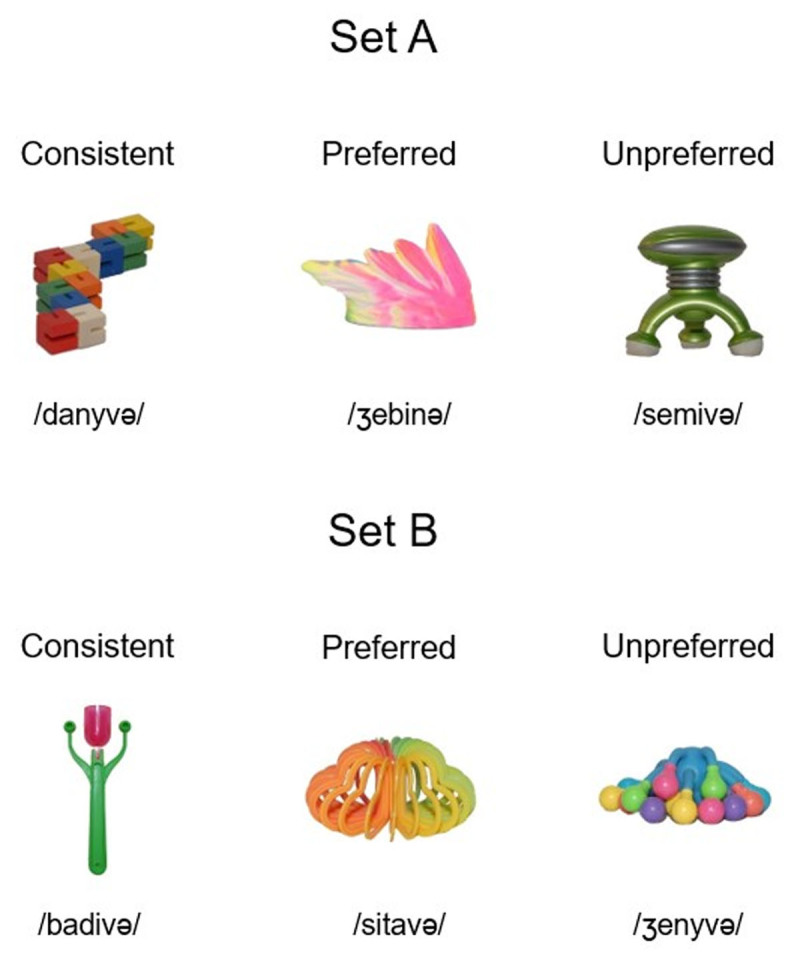
An example object from each set (Set A and Set B) and word group (consistent, inconsistent preferred, and inconsistent unpreferred).

The same 48 pictures of novel objects used in the Jevtović et al. (2022) study and taken from The Novel Object and Unusual Name (NOUN) Database (Horst & Hout, 2016) were selected as novel objects participants were trained on. Half of the pictures were assigned to the words from the Set A and half to the Set B (see Figure 1). Within each set, pictures were randomly assigned the names of the objects and were kept constant for all participants.

### 2.3. Procedure

The experiment was organised in two one-hour long online sessions with a 10–14 day break in between them. At the beginning of each experimental session participants heard an audio message telling them to put on their headphones and adjust the volume to a comfortable level before starting the experiment. They were also instructed to complete each session in one take, in a quiet place avoiding any distractions.

During the first session, participants completed a total of five tasks always presented in a fixed order. Namely, they first completed a pre-test spelling task in which their spelling preferences for all novel words were assessed. To hide the preferred spellings manipulation participants then completed two linguistic distractor tasks (i.e., first a lexical decision and then a real word spelling task). Finally, they completed two tasks assessing their phonological skills: a phoneme deletion task and a nonword repetition task (to measure participants’ phonological short-term memory skills). Only the pre-test spelling task and the nonword repetition task were relevant for the present study. The other tasks were conducted for a different project, and will therefore not be discussed further.

Session 2 started with the phonological training phase during which participants were trained on the names of 24 novel objects. After the training phase, they completed a short non-linguistic distractor task (i.e., the Simon task; Simon, 1969), followed by the self-paced reading task. In the reading task, participants were presented with the spellings of the 24 words they had been trained on as well as 24 words from the untrained set. Finally, at the end of Session 2, participants completed a short picture naming task in which they were prompted to type the names of the presented pictures.

The phonological training and the self-paced reading were programmed using OSWeb online runtime, a JavaScript implementation of OpenSesame 3.3.2 software (Mathôt, Schreij, & Theeuwes, 2012). The pre-test spelling, the nonword repetition task, as well as the picture naming task, were programmed with jsPsych (Version 6.1.; de Leeuw, 2015). To record verbal responses during the nonword repetition task, a modified version of the custom audio recording plugin developed by Vogt, Hauber, Kuhlen, and Rahman (2021) relying on Recorder.js (Diamond, 2016) was used. All tasks were presented to participants through JATOS testing server (Lange, Kühn, & Filevich, 2015).

#### 2.3.1. Pre-test spelling task

Through the pre-test spelling task we assessed each participant’s personal spelling preferences. This was done so as to present inconsistent preferred words in each participants’ preferred spelling and inconsistent unpreferred words in participants’ not preferred spelling in the self-paced reading task (Session 2). For instance, if a participant wrote both /ʒebinə/ and /ʒevabə/ with the letter <g> in the pre-test (Session 1), they would see the spellings <gébine> (preferred) and <jévabe> (unpreferred) in the reading task (Session 2).

A total of 96 novel words (48 target and 48 fillers) were presented. Among the 48 target words, 24 belonged to the Set A and 24 to the Set B. All filler words started with the same sounds as the target words (i.e., the number of consistent and inconsistent words was the same across target and filler words; see Appendix A). Filler words were added to ensure that participants would not remember, two weeks later during the phonological training session, that they had already been presented with all the novel words during Session 1.

Words were aurally presented one by one in a randomised order and the task was self-paced. Each trial started with the recording of the word after which the instruction “Please spell the word you just heard” and a text box prompting participants to spell the word appeared. Upon writing their response, participants either clicked on the indicated button below the text box or pressed ‘enter’ on their keyboard to hear the next pseudoword. The entire task took participants around 10 minutes to complete.

#### 2.3.2. Nonword repetition task (backward and forward)

The aim of the nonword repetition task was to assess participants’ phonological short-term memory capacity in order to explore its relation with generating orthographic skeletons in remembering novel words’ spellings. In the nonword repetition task, participants were presented with sequences of monosyllabic French-like nonwords that they first had to repeat in the same (forward repetition block) and then reversed (backward repetition block) order of the presentation. Namely, in the forward repetition block participants had to repeat the nonwords starting from the first until the last nonword of the sequence, whereas in the backward repetition block those same sequences had to be repeated in the reversed order of presentation (i.e., starting from the last and going to the first nonword from the sequence). The order of the block was fixed (the forward block was always followed by the backward repetition block) and both blocks started with a sequence containing two nonwords. The sequences gradually increased in length, from two to eight, and two sequences of each length were presented (i.e., two sequences of two nonwords, two sequences of three nonwords, etc.). To make sure that nonwords within each list sound as phonologically distinct as possible from all the other nonwords within the same sequence, they all contained a different vowel sound, and none started or ended with the same consonant (see Appendix B for the complete list of nonwords used in this task).

Each trial had the following structure. First, a fixation cross appeared at the centre of the screen. After 1000 ms it was replaced by a picture of an ear, indicating that the listening part of the trial was in course and at the same time the sequence of nonwords was played. In each sequence, there was a 750 ms pause between any two nonwords. After the last nonword of the sequence had been played, the picture of the ear disappeared and there was a 500 ms pause before the picture of a mouth appeared initiating the microphone and the production part of the trial. Participants had 10 seconds to respond before the picture of the mouth disappeared indicating the end of that trial. Participants had to click on the button presented at the centre of the screen to start the next trial and hear the next sequence. At the beginning of each block two practice examples were completed. During the practice trials participants were prompted to record their response and then compare their response to the correct one that was played to them after they had given their response. The entire task took around 20 minutes to complete.

#### 2.3.3. Phonological training phase

Session 2 started with the phonological training phase during which participants were trained on the pronunciations of 24 words from one of the two sets (Set A or Set B). The two sets were counterbalanced across participants as half of them were trained on the Set A and half on the Set B.

At the beginning of the task, participants were informed that they needed to learn the names of the objects they would be presented with as their performance would afterwards be tested. In addition, they were explicitly told that the names of all objects were masculine. This was done to make sure participants would internalize the masculine gender of the nouns during the learning phase and thus not be surprised to see masculine pronouns in the consequent reading task.

**Figure 2:**
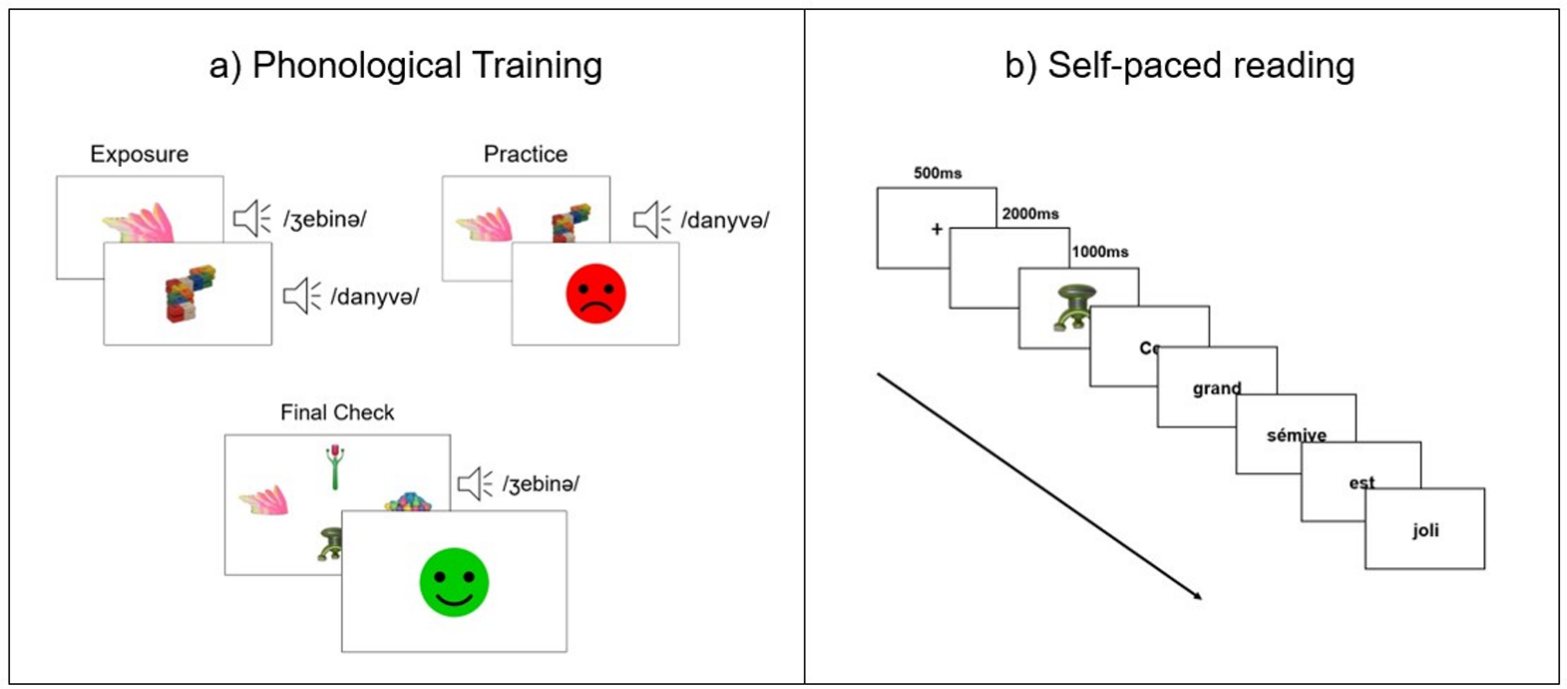
Structure of the phonological training (on the left) and the self-paced reading task (on the right).

The entire phonological training was divided into two parts. The first part consisted of four training blocks with an identical structure (see Figure 2a). In each block, participants were trained on the names of 6 objects (two consistent and four inconsistent) and the order of the four practice blocks was counterbalanced across participants. Each block started with an *exposure phase*. During the exposure phase, pictures of the 6 objects were presented one by one at the centre of the screen. While the picture of the object was on the screen its name was played three times in a row at different speeds (i.e., /bemanə/ -> /be/ – /manə/ -> /bemanə/). Once all 6 objects had been seen together with the auditory display of their name, participants completed the *practice phase* (see Figure 2a). During the practice phase, participants were presented with pictures of two objects, one on the left and one on the right side of the screen, and at the same time, the name of one of them was played. Participants’ task was to indicate, by pressing ‘A’ for the object on the left and ‘L’ for the object on the right on their keyboard, to which of the two objects corresponded the name they just heard. After each response, participants received a feedback message (in the form of a happy or sad face) indicating whether they had responded correctly or not. Each picture was paired with every other picture and appeared once on the left and once on the right side of the screen, giving this way a total of 60 trials in each practice block. At the end of each practice block, participants received feedback on their overall accuracy (in %).

At the beginning of the second part of the phonological training, the names and the pictures of all 24 objects were played once again. Pictures were randomly presented one by one at the centre of the screen together with the auditory presentation of their names, and participants moved from one picture to the next one by pressing ‘enter’ on their keyboard. Next, participants performed the *final check phase*. In this phase, participants were presented with pictures of four objects, one on the left, right, top, and bottom, areas of the screen (see Figure 2a) and at the same time they heard the name of only one of them. The arrows on the keyboard were used to respond to which of the four objects corresponded to the name that was played (left, right, up, down). As in the previous practice trials, participants were informed immediately after responding whether their response was correct. To ensure that each picture appeared an equal number of times at each of the four positions on the screen and was paired an equal number of times with every other picture, a Latin square design with a total of 144 trials was employed for counterbalancing position and pairing of the pictures. Finally, participants were informed about their overall performance at the end of the task.

#### 2.3.4. Self-paced reading

In the self-paced reading task participants were presented with the spellings of the 24 words from the set they had been trained on (*trained words*) along with the spellings of the 24 words from the untrained set (*untrained words*). All words were embedded in eight four-to-seven-word long sentences (see Table 3) presented word by word at the centre of the screen (see Figure 2b). To make sure each word (both trained and untrained) appeared the same number of times in each of the six possible positions in the sentence (two to seven), the position of the target word in the sentence was counterbalanced across participants using a Latin square procedure. Along with varying sentence length, counterbalancing position in the sentence where the target words appeared was done to ensure participants would not anticipate the appearance of the target word and press ‘enter’ without reading it.

**Table 3:** Sentences from the self-paced reading task and their English translations.


FRENCH SENTENCES	ENGLISH SENTENCES

Ce **xxx** est petit	This **xxx** is small

Ce grand **xxx** est joli	This big **xxx** is pretty

Ceci est un **xxx** gigantesque	This is one big **xxx**

Ceci est un petit **xxx** magnifique	This is one small magnificent **xxx**

Cet objet est un **xxx** minuscule	This object is one very small **xxx**

Cet objet est un petit **xxx** magnifique	This object is one small magnificent **xxx**

Ce grand objet est un **xxx** magnifique	This big object is a magnificent **xxx**

Ce grand objet est un magnifique **xxx**	This big object is one magnificent **xxx**


*Note*: Bold exes represent the place where target words appeared. Due to syntactic differences across languages, the position of the target word differs between French sentences and their English translations. * French sentences were matched with Spanish sentences used in Jevtović et al. (2022) study on the length as well as the place where the target word appeared.

Each sentence was preceded by a picture of the object whose name was to appear in that sentence, in order to prime the name of the object in the participant’s mind. Trained trials were preceded by a picture of a trained object (i.e., the picture associated with the novel word to appear in the sentence), and in the untrained trials, participants were shown an object that has been associated with the word from the untrained set. Participants were instructed to read each sentence as fast as possible without making pauses on any word. Sentences were presented in randomized order. At the beginning of each trial, a fixation cross with a duration of 500 ms appeared at the center of the screen. Next, the picture of the object was presented on the screen for 2000 ms. The picture was replaced with a blank screen presented for 1000 ms, after which the first word of the sentence appeared at the center of the screen. Words were then presented one by one, and the task was self-paced. Namely, to advance from one word to the next one, participants had to press ‘enter’ on their keyboard. After reading the last word of the sentence, the next trial was initiated by pressing ‘enter’ (see Figure 2b). Before starting the task, participants were presented with three practice sentences preceded by three known objects (e.g., a book, a glass, and a pencil) and shown two times each. Reading times, measured from the moment the word appeared at the center of the screen until participants pressed enter, were recorded for all words. The entire task took approximately 10 minutes to complete.

#### 2.3.5. Picture naming task

In the picture naming task participants were presented with the pictures of the 24 objects they had been trained on and were instructed to type their names. Pictures were presented one by one at the centre of the screen in a randomised order and the exact structure of the trial was the following: first, the picture of an object appeared at the centre of the screen. After 1000 ms the picture became smaller, and a text box appeared below it, prompting participants to type in the name of the object shown in the picture. After giving their response, participants either pressed the indicated button below the text box or ‘enter’ on their keyboard to see the next picture. The task took around five minutes to complete.

### 2.4. Data pre-processing and analysis

Results from the pre-test spelling task were used to determine each participant’s individual spelling preferences in order to present inconsistent words in their preferred or unpreferred spellings. Pre-test spelling data are shown in Appendix C but will not be further discussed as doing so is beyond the scope of the present paper. Similarly, data from the phonological training task served as an indicator of whether participants acquired the novel words they had been trained on, and hence exclude those below the set criteria (<70% of accuracy). Consequently, only descriptive statistics for the phonological training are included. Finally, in the nonword repetition task, a total number of correctly repeated nonwords in both the forward and the backward block was calculated for each participant and this score was used as an indicator of participants’ PSTM capacity (see Section 3.3.).

#### 2.4.1. Self-paced reading task

Reaction times (RTs) for the target words (trained and untrained) from the self-paced reading task were analysed using linear mixed effects models (Baayen, Davidson, & Bates, 2008) in the R statistical environment (Version 4.0.2; R Core Team, 2020) through RStudio (Version 2022.2.0.443; RStudio Team, 2020). The analysis was performed using the *lme4* package (Version 1.1–23; Bates, Mächler, Bolker, & Walker, 2015) and the *p* values were obtained through the *lmerTest* package (Version 3.1–2; Kuznetsova, Brockhoff & Christensen, 2017) which uses the Satterthwaite’s degrees of freedom method. Data wrangling and visualisation was done using the *tidyverse* collection of R packages (Wickham et al., 2019). To build the random effects structure, a data-driven approach was taken (Bates et al., 2015). Consequently, all reported models include the maximal random effects structure for all experimental manipulations of interest supported by the data.

Before analysing RTs, extreme values (RTs below 150 ms and above 2500 ms; eight data points in total) were removed. In line with the Box-Cox test (Box & Cox, 1964), RTs were then log transformed. Outlier removal was based on scaled residuals (Baayen & Milin, 2010) and the models reported here are refitted after removing absolute values of residuals greater than 3 (1.18% of all data points were removed this way).

To test whether there is a significant difference in reading words with a unique spelling (i.e., consistent words) and each of the two inconsistent spellings (i.e., inconsistent preferred and inconsistent unpreferred words), the three level factor *Group* was deviation coded (Schad et al., 2020).^3^ The first contrast thus compared the RTs between consistent and inconsistent preferred words (hereafter *ConsistentVsPreferred*; consistent words coded as –0.33, inconsistent preferred as 0.67 and inconsistent unpreferred as –0.33) while the second contrast compared consistent to inconsistent unpreferred words (hereafter *ConsistentVsUnpreferred*; consistent words coded as –0.33, inconsistent preferred as –0.33 and inconsistent unpreferred as 0.67). Factor *Training* (trained vs untrained words) was initially deviation coded (trained as –0.5 and untrained as 0.5). In the case of significant interactions, it was then treatment (dummy) coded in order to change the reference level (trained or untrained coded as 0) and look at the differences of interest at only one level of training (either trained or untrained). Finally, the factor *Set* referring to two sets of items, was deviation coded (Set A as –0.5 and Set B as 0.5) and included in the model as the fixed covariate.

Additionally, in order to explore the relationship between generating orthographic skeletons for newly acquired spoken words and later recall of those same words, a score indicating an individual’s tendency to generate orthographic skeletons was calculated for all participants. This score was operationalized as a difference in mean reading times between inconsistent unpreferred and consistent trained words and was denoted as the *orthographic skeleton effect (OSE)*. Higher positive values indicate a larger difference between reading words in unpreferred and unique spellings thus indicating a stronger surprisal effect in reading aurally acquired words that do not match participants’ expectations. The OSE score was, along with the phonological short-term memory score from the nonword repetition task (i.e., the PSTM score), used to predict the accuracy in the final picture naming task in the multiple linear regression analysis (see Section 3.3.)

#### 2.4.2. Picture naming task

In the picture naming task, participants had to spell the names of the 24 objects they had been trained on by typing their names. To obtain a more precise measure of word recall regardless of the spelling preference, instead of calculating accuracy as a binary 1 (correctly spelled word in its entirety) or 0 response (incorrectly spelled word regardless of its phonetic similarity to the correct response), phonetic distance between the correct and given response was calculated for each participant’s responses using the ALINE string alignment algorithm.^4^ The ALINE algorithm quantifies the phonetic similarity between two strings. It can be considered a complementary measure to Levenshtein distance, given that apart from the number of insertions, deletions and substitutions, it also takes into account phonetic features in calculating the distance between two strings (Kondrak, 1999). ALINE distance was calculated for each response through *alineR* package in R (Downey, Sun, & Norquest, 2017) which uses the ALINE algorithm to calculate the phonetic similarity score between any two word strings and gives a finite value from 0 to 1. Finally, given that in *alineR* the phonetic similarity score between the two identical strings results in 0 (no differences), meaning that higher values indicate lower accuracy, for the sake of simplicity we report the Inverse ALINE score calculated as 1-ALINE (i.e., higher values stand for higher accuracy and better recall). Note that since 1-ALINE is indexing phonetic closeness, the two plausible spellings of an inconsistent word would both give a score of 1.

## 3. Results

### 3.1. Phonological training

The accuracy in all four blocks as well as the final check phase of phonological training was close to ceiling (see Table 4). Importantly, there were no differences between the two sets of words in the final check phase (Set A: M = 93, SD = 7.08; Set B: M = 93.8, SD = 6.37; *t*(44) = .394, *p* = .695).

**Table 4:** Mean Percentage of Accuracy (SDs) per Training Block and in the Final Check Phase.


	BLOCK1	BLOCK2	BLOCK3	BLOCK4	FINAL CHECK

**Set A**	95.8 (4.64)	96.9 (2.49)	97.1 (4.29)	95.6 (4.61)	93 (7.08)

**Set B**	96.4 (4.55)	98.1 (3.19)	95.5 (7.35)	95.5 (5.69)	93.8 (6.37)


#### 3.2. Self-paced reading task

In the self-paced reading task participants were presented with the spellings of aurally acquired novel words. Words from the consistent group were shown in their unique spellings while inconsistent words were presented in either their preferred (inconsistent preferred group) or unpreferred spellings (inconsistent unpreferred group).

**Figure 3:**
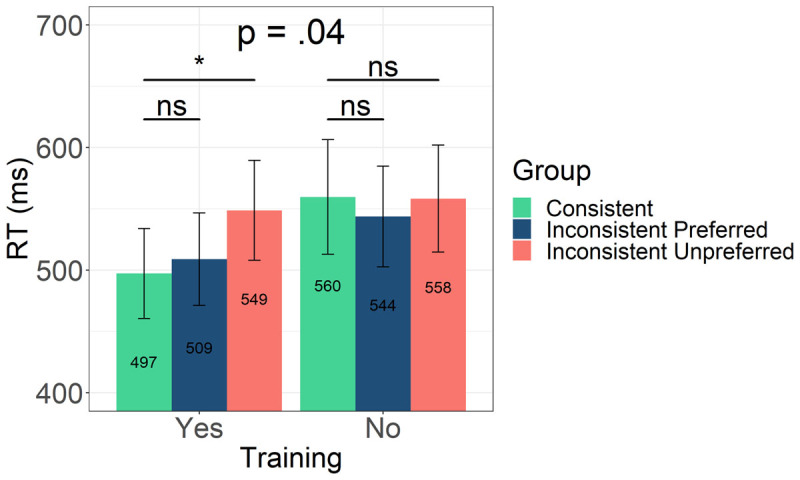
RTs from the self-paced reading task per group of words. *Note*: Consistent words are shown in green, inconsistent preferred in blue, and inconsistent unpreferred in red. Trained words are presented on the left and untrained on the right. Error bars represent the standard error of the mean (SEM).

The overall model looking at the main effects of *Training, ConsistentVsPreferred* and *ConsistentVsUnpreferred* contrasts, as well as the interactions between *Training* and each of the two contrasts, included by-participants and by-item random intercepts, by-participants random slopes for *Training* and *ConsistentVsUnpreferred* contrast, as well as by-item random slopes for *Training* (see Table 5). The model showed a significant main effect of *Training* (*β* = 0.044, *SE* = 0.020, *t* = 2.24, *p* = 0.031) with trained words (M = 518, SD = 255) yielding shorter RTs than untrained words (*M* = 554, *SD* = 291). Neither the *ConsistentVsPreferred* (*β* = 0.002, *SE* = 0.027, *t* = 0.082, *p* = 0.935) nor the *ConsistentVsUnpreferred* (*β* = 0.073, *SE* = 0.028, *t* = 1.22, *p* = 0.227) were significant. However, the interaction between *Training* and *ConsistentVsUnpreferred* contrast was significant (*β* = –0.077, *SE* = 0.037, *t* = –2.05, *p* = 0.046; see Figure 3).

**Table 5:** Fixed and Random Effects Structure of the Overall Model.


FIXED EFFECTS	β	SE	*t* VALUE	*p*

**(Intercept)**	6.12	0.068	90	0.00***

**Training**	0.044	0.020	2.24	0.031*

**ConsistentVsPreferred**	0.002	0.027	0.082	0.935

**ConsistentVsUnpreferred**	0.035	0.028	1.22	0.227

**Set**	0.211	0.135	1.56	0.125

**Training: ConsistentVsPreferred**	–0.034	0.037	–0.917	0.364

**Training: ConsistentVsUnpreferred**	–0.077	0.037	–2.05	0.046*

**RANDOM EFFECTS**		**VARIANCE**	**STD.DEV.**	

**Item: (Intercept)**		0.003	0.059	

**Item: Training (slope)**		0.002	0.044	

**Participant: (Intercept)**		0.206	0.453	

**Participant: Training (slope)**		0.007	0.086	

**Participant: ConsistentVsUnpreferred (slope)**		0.004	0.059	


*Note*: Asterisk denotes statistical significance: ∗ *p* < 0.05; ∗∗ *p* < 0.01; ∗∗∗ *p* < 0.001. * The exact structure of the model was the following: logRT~1+Training* ConsistentVsPreferred + Training* Consistent Vs Unpreferred + Set + (1+Training+ ConsistentVsUnpreferred ||participant) + (1+Training ||item).

Follow-up analysis (performed by treatment coding the factor training) looking at RTs first only in the group of trained words and then only within untrained words, showed that while the *ConsistentVsUnpreferred* difference was significant for trained words (*β* = 0.078, *SE* = 0.032, *t* = 2.25, *p* = 0.028) the same comparison was not significant when only untrained words were considered (*β* = –0.004, *SE* = 0.034, *t* = –0.109, *p* = 0.914). The *ConsistentVsPreferred* difference was not significant within the trained (*β* = 0.019, *SE* = 0.031, *t* = 0.616, *p* = 0.539) or the untrained words (*β* = –0.015, *SE* = 0.033, *t* = –0.451, *p* = 0.654; see Figure 3).

In sum, the analysis of RTs in the self-paced reading task shows that first, trained words were read overall faster than untrained ones, thus indicating a significant aural training advantage when reading novel words acquired through aural instruction. Secondly, and importantly for the predictions of the study, within trained words, no differences were found between consistent and inconsistent preferred words. Inconsistent unpreferred words however, yielded significantly longer reading times.

##### 3.2.1. Further investigation of the training effect

To further inspect the training effect found both in the present as well as the study conducted with Spanish speakers (Jevtović et al., 2022), two additional models (one per language) compared trained and untrained words for each spelling condition separately. To that end, three contrasts of interest were set: the first one compared trained and untrained consistent words (i.e., *ConsistentSpelling* contrast; consistent trained coded as –0.5, consistent untrained coded as 0.5, the rest coded as 0), the second one compared trained and untrained preferred words (i.e., *PreferredSpelling* contrast; preferred trained coded as –0.5, preferred untrained coded as 0.5, the rest coded as 0), and the third one compared trained and untrained unpreferred words (i.e., *UnpreferredSpelling* contrast; unpreferred trained coded as –0.5, unpreferred untrained coded as 0.5, the rest coded as 0).

The model looking into data from French speakers included by-participants and by-item intercepts as well as by-participant random slopes for *PreferredSpelling* contrast. The model showed a significant RT difference between trained and untrained consistent words (*β* = 0.081, *SE* = 0.024, *t* = 3.35, *p* = 0.001). The same difference for preferred spellings however failed to reach the level of significance (*β* = 0.047, *SE* = 0.025, *t* = 1.89, *p* = 0.065). Finally, no differences in RT were found between trained and untrained unpreferred words (*β* = 0.004, *SE* = 0.024, *t* = 0.175, *p* = 0.861).

The model looking into data from Spanish speakers included by-participants and by-item intercepts as well as by-participant random slopes for *ConsistentSpelling* and *UnpreferredSpelling* contrasts. The model showed no RT differences between trained and untrained consistent (*β* = –0.019, *SE* = 0.027, *t* = –0.694, *p* = 0.491) or preferred words (*β* = –0.036, *SE* = 0.025, *t* = –1.46, *p* = 0.144). However, a significant difference was found between trained and untrained unpreferred spellings (*β* = –0.063, *SE* = 0.030, *t* = –2.09, *p* = 0.042).

### 3.3. Exploratory analysis: the relationship between generating orthographic skeletons and phonological short term memory in novel word recall

To investigate the relation between phonological short-term memory (PSTM), and individual tendency to generate orthographic skeletons (OSE) in novel word recall, a multiple regression with Inverse ALINE score as a dependent variable was performed. Due to technical issues (i.e., their microphone not working) three participants failed to complete the nonword repetition task. Therefore, the multiple regression analysis was performed on data from 43 participants.

Two predictors and their interaction were included in the model: PSTM score, measured as the total number of correctly repeated pseudowords in both the forward and the backward nonword repetition task (range: 34 to 122) and the OSE score, calculated as the difference between reading aurally acquired words with unpreferred and those with unique spellings (range: –217 ms to 475 ms). Both the PSTM score and the OSE score were standardized before running the model. The collinearity between factors was checked with VIF.mer (Frank, 2011) and all VIFs were below 2. Finally, the model with the best fit (i.e., the model with the highest adjusted R-squared value) was the one that included both predictors along with their interaction.

**Figure 4:**
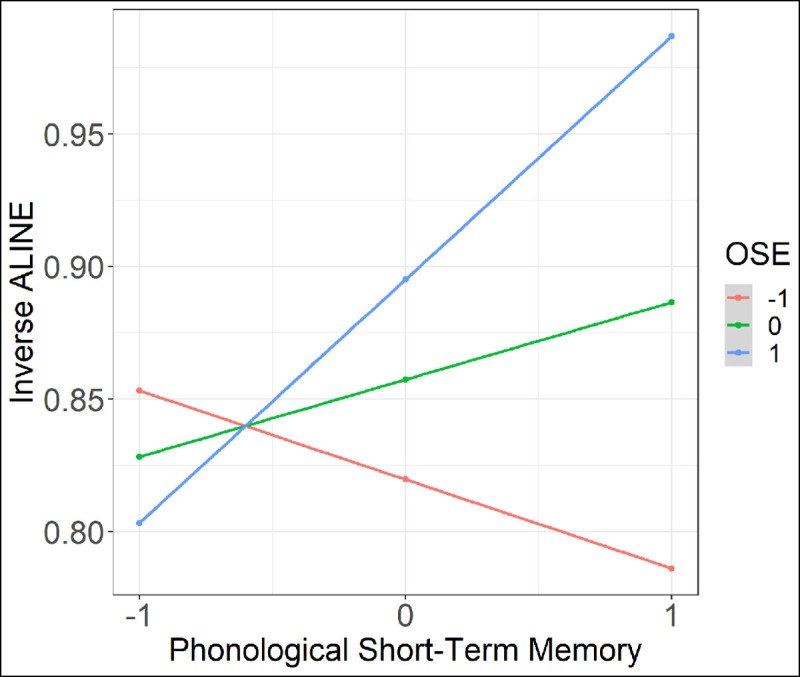
Visual representation of the interaction between PSTM and OSE. *Note*: The relationship between the OSE and novel word recall (Inverse ALINE similarity score) modulated by PSTM capacity. The graph shows that, as PSTM score increases, the positive link between OSE and word recall increases. Both predictors (PSTM and OSE) are represented as z-scores.

The results of the regression indicate that the two predictors along with their interaction predicted 28.8% of the variance in the picture naming task (*R^2^* = .28, *F*(3,39) = 5.12, *p* <.01). While OSE index significantly predicted the inverse ALINE score (*β* = 0.038, *SE* = 0.018, *t* = 2.07, *p* = 0.045), indicating that higher values of OSE lead to better recall (i.e., a higher score in the picture naming task), the PSTM score failed to reach the level of significance (*β* = 0.029, *SE* = 0.015, *t* = 1.96, *p* = 0.057). However, there was a significant interaction between the two (*β* = 0.063, *SE* = 0.024, *t* = 2.58, *p* = 0.014). The interaction indicated that the relationship between one predictor variable and the outcome variable is modulated by the other predictor. In this particular case, the interaction shows that the positive link between the OSE and the inverse ALINE score is observed in participants with higher PSTM scores (see the partial plot presented in Figure 4 for the visual representation of the significant interaction between the predictors).

## 4. Discussion

The main goal of the present study was to test whether orthographic skeletons (i.e., preliminary orthographic representations) for novel words with two possible spellings are generated even in a language with a high degree of sound-to-spelling inconsistency. To that end, French adults first acquired novel words via aural training and were then exposed to novel words’ spellings in a self-paced reading task. Overall, the results show that French speakers generated orthographic skeletons for newly acquired spoken words, even when more than one spelling was possible. This conclusion is supported by several findings: Firstly, trained words were read overall faster as compared to the untrained ones, thus yielding a significant aural training advantage. Secondly, no differences in reading times were found between words with unique and those presented in their preferred spellings. By contrast, inconsistent words presented in their unpreferred spellings led to significantly longer reading times. This implies that there was a mismatch between the orthographic skeletons participants had generated and the spellings they were later presented with. Finally, no significant differences in reading times were observed between different spellings of untrained words. We take this as evidence that orthographic skeletons for words with more than one spelling are generated even in a language with a high degree of sound-to-spelling inconsistency. By using the same design as the one previously used with speakers of a transparent language (i.e., Spanish; Jevtović et al., 2022), the findings from the present study confirm the original findings reported by Wegener and colleagues (2018; Wegener et al., 2020).

Languages vary in how their phonology is represented in written language. In transparent languages such as Spanish, the relationship between sounds and letters is relatively simple as most sounds map onto only one letter and vice versa. Orthographic skeletons generated in a transparent language will – in the majority of cases – match the actual spelling. As they are usually confronted with spellings in line with their predictions, speakers of Spanish may be strongly prone to generate orthographic skeletons regardless of the number of possible spellings. In opaque languages like French however, the relationship between sounds and letters is more complex. Due to many sounds having multiple grapheme representations (Ziegler et al., 1996), generating a spelling expectation for a newly acquired spoken word comes with a high risk of generating an incorrect representation. Given the higher probability of generating an incorrect spelling expectation, the process of generating orthographic skeletons for aurally acquired words containing inconsistent sounds, and consequently multiple legal spellings, may be supressed in speakers of opaque languages. The present data suggest that it is not the case as the same pattern of results was observed in both Spanish (Jevtović et al., 2022) and French skilled readers (present data). Both studies report significantly longer reading times only for inconsistent words presented in their unpreferred spellings, while no difference between reading preferred and unique spellings were found. This shows that French speakers are not affected by the overall complexity of sound-to-spelling mappings when generating orthographic skeletons. Moreover, the present study also confirms and expands on the findings observed in English (Wegener et al., 2018; Wegener et al., 2020; see also Beyersmann et al., 2021). In two studies, Wegener and colleagues showed that English-speaking children were faster to read trained as compared to untrained novel words. Importantly, the facilitation was driven by faster reading times found for predictable as compared to unpredictable spellings. As the latter had multiple spelling options, it remained unclear whether orthographic skeletons in an opaque language would be generated even when, due to multiple options, the spelling of the novel spoken word is uncertain. By controlling the number of inconsistent spellings, we show that orthographic skeletons for words with multiple, and hence unpredictable, spellings are generated even when the overall inconsistency of the language is high.

**Figure 5:**
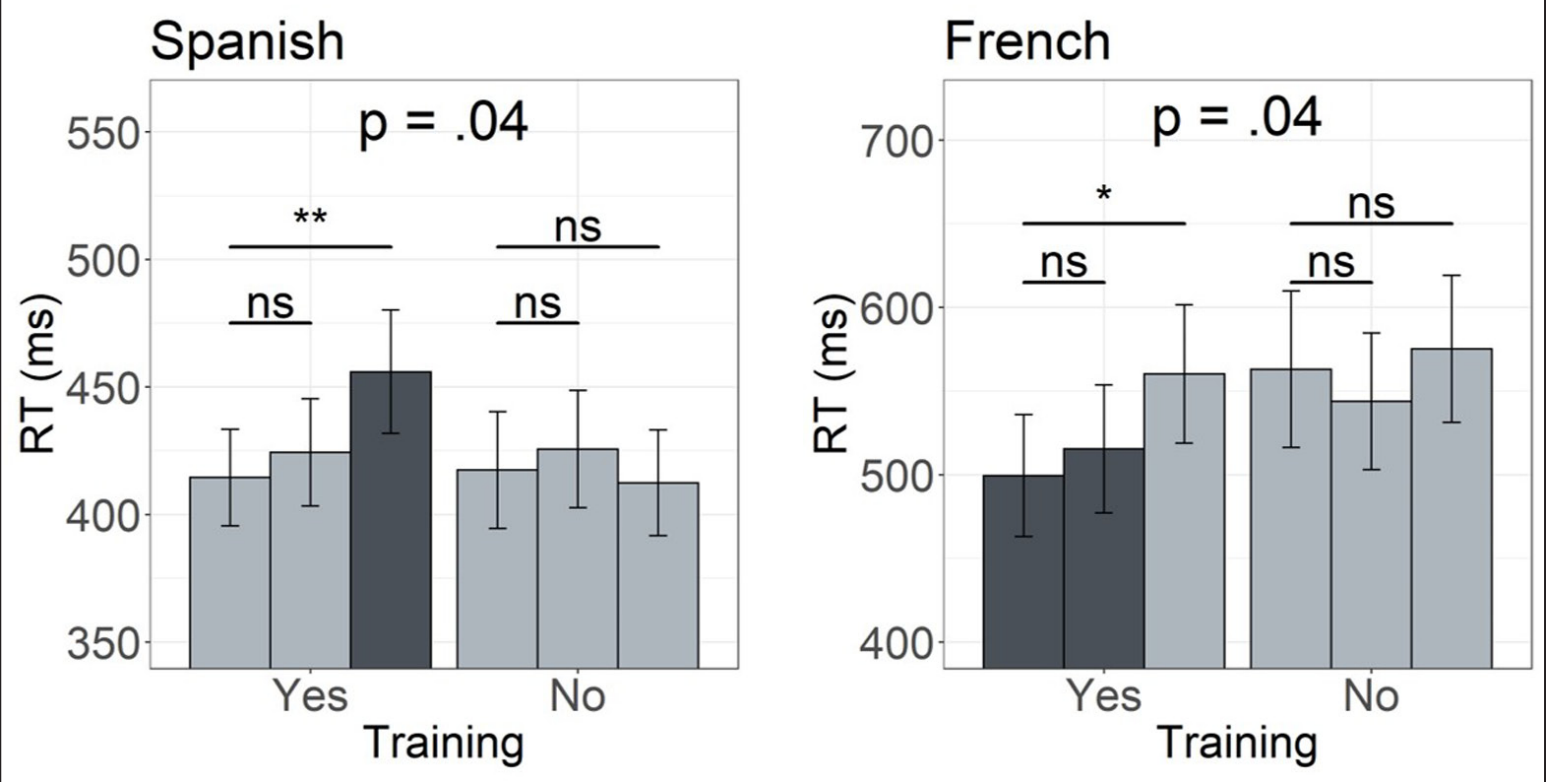
The pattern of results observed in Spanish (left) and French readers (right). *Note*: In both graphs the first bar in each training group represents consistent words, the second one inconsistent preferred and the third one represents inconsistent unpreferred words. * Spanish data used to create the figure were obtained from https://osf.io/h69dg/.

A surprising difference between the current findings and those reported by Jevtović et al. (2022) is a significant *aural training advantage* observed in the present study but not in the one with Spanish speakers. In the present study participants were overall faster to read trained as compared to untrained words. This advantage seems to be driven by faster reading times for previously acquired words with consistent and those presented in the preferred of the two possible spellings.^5^ The evidence for the orthographic skeleton hypothesis is thus found in the *facilitatory effect* present when reading previously acquired words in line with predicted spellings (see Figure 5). This is in line with previous research showing facilitatory effects of aural training on subsequent word reading, and in particular, the formation of novel orthographic representations (e.g., Álvarez-Cañizo, Suárez-Coalla, & Cuetos, 2019; Johnston et al., 2004; McKague, Pratt, & Johnston, 2001, Wegener et al., 2018, Wegener et al., 2020). The same was however not the case in Spanish, where consistent and inconsistent preferred words did not differ in reading times across the two training conditions. It was the inconsistent words shown in their unpreferred spellings that yielded longer reading times as compared to the untrained words, thus resulting in an *aural training disadvantage* for words not matching participants’ expectations (Jevtović et al., 2022). Evidence for the orthographic skeleton hypothesis in Spanish is thus found in the *inhibitory effect* present when reading previously acquired words not in line with predicted spellings (see Figure 5).

The absence of the training advantage in Spanish readers was explained by the overall transparency of the Spanish language system (Jevtović et al., 2022). As already discussed, speakers of a transparent language such as Spanish are used to encountering spellings in accordance with their predictions. Seeing words that do not match their predictions could thus yield a surprisal effect and lead to longer reading times. Conversely, in opaque languages such as French and English, readers are more rarely confronted with correctly predicted spellings. The surprisal effect may therefore be manifested as a significant facilitation for words that do match the predicted spellings. This is indeed in line with the pattern of results observed in the original study by Wegener et al. (2018). These studies thus show that generating orthographic skeletons when there is a risk of error (i.e., for words with more than one spelling) is done in both transparent and opaque languages. However, the consequences of generating orthographic skeletons for word reading may be manifested differently depending on the overall consistency of the language. While generating orthographic skeletons in opaque languages (English and French) comes with a *reading benefit*, generating them in transparent languages (Spanish) seems to come with a *reading cost* when predictions are not met. Replicating these findings in other transparent and opaque languages is however needed to draw any strong conclusions regarding the cross-linguistic differences observed so far.

In addition to showing that generating orthographic skeletons has consequences for *word reading*, the present study reveals differences in *word learning* related to individual tendency to generate orthographic skeletons. Participants who were more prone to generate orthographic skeletons in the present study were also better at recalling novel words at the end of experiment. Importantly, this positive link was modulated by individual phonological short-term memory (PSTM) capacity, since the positive correlation between the tendency to generate orthographic skeletons and later word recall was observed in individuals with higher PSTM scores. This interaction thus suggests that only participants with average-to-high PSTM score were able to benefit from generating orthographic skeletons during aural training. The previously reported decline in the positive link between PSTM skills and spoken word learning occurring at the age of 8 (Gathercole, Willis, Emslie, & Baddeley, 1992; Gathercole et al., 1994), may thus be partly related to the emergence of the mechanism which maps sounds-to-letters and this way generates preliminary orthographic representations. In line with the reading time data, this finding shows that generating orthographic skeletons comes with a benefit, in this case, a *word learning benefit*. This further shows that in skilled readers, orthography affects spoken word learning even when not present during the learning process. Finally, these findings revealing individual differences in generating orthographic skeletons show that speakers vary in how much they rely on orthography when acquiring novel vocabulary via aural instruction. Future research with larger sample size is needed to understand better the interplay between different mechanisms available at different stages of development that aid novel word learning as well as individual differences in relying on those same mechanisms.

By controlling for the number of possible, or dominant spellings, both the present study and the one conducted by Jevtović et al. (2022) show that orthographic skeletons are generated even when there is uncertainty regarding a novel word’s spelling. However, an important limitation of these studies is that neither of the two is able to answer whether orthographic skeletons are generated for words with *more than two* spellings. Indeed, in both studies, in order to control for the number of possible or dominant spellings, inconsistent words had only two possible spellings. Some evidence that orthographic skeletons are generated even for words with *more than two* possible spellings may however be found in the present study. Although we tried to create novel words with a unique or only two possible spellings – and all participants wrote the words using one of the two planned inconsistent spellings in the pre-test spelling task – some words (both consistent and inconsistent) could potentially have more than two spellings. By doubling the second or the third consonant some of the words have potentially more than two legal orthographic forms in French (e.g., /beman/ as <bemanne> or <bemmane>). Replicating the findings observed in Spanish, however, excludes the possibility that this confound (due to the nature of the French writing system) affected the results. At the same time, this property of the French writing system provides some evidence that orthographic skeletons are generated even when multiple spellings are possible. Another limitation is related to the design used in both this as well as Jevtović et al. (2022) study. To assess individual spelling preferences, participants were asked to spell out all target words (i.e., both trained and untrained) two weeks before the learning phase. Despite adding filler pseudowords, additional filler tasks, and a two-week delay between the two experimental sessions were added, this pre-exposure to novel words may have influenced the observed results. The fact that significant differences between consistent and inconsistent unpreferred words were found only in the group of trained but not the untrained words, however, speaks against such a possibility. Nevertheless, future studies could prolong the time delay between the two sessions or add even more distractor tasks. Alternatively, to reduce the potential influence of previous orthographic exposure, spelling preferences could be provided orally in future studies.

Another methodological limitation which should be mentioned is related to the nature of the reading task used in the present study. To prime the word appearing in the sentence, and this way activate the orthographic skeleton previously generated, each sentence in the reading task was preceded by the picture of the object whose name was to appear in that sentence. The picture thus served as semantic context. Although priming words’ spellings with pictures was done on purpose, it does not resemble reading happening in a more naturalistic context. The observed effects may therefore be stronger than they would have been in a more ecologically valid reading task. In the original study by Wegener and colleagues (2018) sentences were indeed contextually supportive and mimicked more natural reading. Nevertheless, even though picture priming may have magnified the observed effects, recent evidence shows that orthographic skeleton effects hold even in the absence of any contextual support, that is, when novel words are read in isolation (see Wegener et al., 2022). Interestingly, the fact the effects of aural training on novel word reading are present in a variety of reading tasks points to a robust nature of the orthographic skeleton effect.

In sum, the present findings show that orthography plays a role in spoken word learning. Skilled readers generate preliminary orthographic representations in the absence of orthography even when there is risk of error. This raises a possibility that generating orthographic skeletons during spoken word learning may be automatic and unconscious. However, the automaticity of the phenomenon needs to be investigated in studies comparing explicit and implicit word learning. Altogether, the findings of the present study are in line with broader research showing pervasive effects of orthography on spoken language processing (Castles, Holmes, Neath, & Kinoshita, 2003; Seidenberg & Tanenhaus, 1979; Ziegler & Ferrand, 1998).

## Data Accessibility Statements

All data, statistical and experimental scripts as well as stimuli material associated with this manuscript are available at: https://osf.io/dmtn4/?view_only=854d125c524a4f7b944576c95c655395.

## Additional File

The additional file for this article can be found as follows:

10.5334/joc.250.s1 Appendices. Appendix A to C.

## Footnotes

Phoneme-to-grapheme and grapheme-to-phoneme mappings refer to mappings between individual sounds and letters while sound-to-spelling and spelling-to-sound refer to higher level mappings (i.e., the entire spoken and written language system).

This criterion along with the sample size were based on the ones reported by Jevtović et al. (2022).

Note that the maximum number of possible contrast for a categorical variable is n–1, n being the number of levels (Schad et al., 2020). Thus when comparing three conditions, as is the case here, there are only two possible comparisons with one common baseline. The most evident baseline in our case are consistent words because the other two are more similar phonologically (two inconsistent groups of words). This is why we compared consistent to inconsistent preferred and consistent to inconsistent unpreferred.

Namely, to avoid a situation in which for example a response <banive> and <dadibe> instead of <bamive> would both result in a value of 0, and one is almost correct while the other is completely incorrect, accuracy is represented as a similarity score indicating how close participants were to the correct spelling.

Note however that when the three groups of words were separately compared across training, no significant difference was obtained for inconsistent preferred words (p = 0.065; see Section 3.2.1.).
