# Supplementary material for: Déjà-lu: When Orthographic Representations are Generated in the Absence of Orthography
Source: J Cogn. 2023 Jan 12;6(1):7. doi: 10.5334/joc.250 (PMC9838226; doi:10.5334/joc.250)
Supplement: Appendices. — Appendix A to C. [file joc-6-1-250-s1.pdf]

Appendix A

Filler pseudowords from the pseudoword spelling task

Table A1

Consistent	Inconsistent	Inconsistent
/bedavə/	/ʒinymə/	/ʒibybə/
/dabineə/	/ʒetivə/	/ʒebabə/
/tubanə/	/sebanə/	/sevivə/
/mavymə/	/sibyvə/	/sidabə/
/nydivə/	/fatavə/	/fanymə/
/vemagə/	/femidə/	/fetogə/
/pivavə/	/kimybə/	/kilydə/
/lydanə/	/ketagə/	/kemumə/
/bamivə/	/ʒimanə/	/ʒipagə/
/dedabə/	/ʒebanə/	/ʒelyvə/
/talynə/	/simamə/	/simymə/
/medivə/	/selidə/	/sedivə/
/nedumə/	/fabigə/	/fadumə/
/vamyvə/	/femydə/	/fetabə/
/pitogə/	/kinynə/	/kilavə/
/labydə/	/kedibə/	/kepymə/

Appendix B

French like monosyllabic nonword used in the nonword repetition task

Table B1

**Practice trials**	/kɛd/	/ʃis/						
	/lɛp/	/fyb/	/mav/					
**2 items sequences**	/tɔʃ/	/nyp/						
	/fik/	/ʁœd/						
**3 items sequences**	/lyz/	/zɑ̃ʃ/	/nɔl/					
	/dyl/	/nɔk/	/zɛt/					
**4 items sequences**	/suv/	/zɔd/	/ʃym/	/nɛl/				
	/nys/	/tup/	/mab/	/fɛʃ/				
**5 items sequences**	/sod/	/vɛk/	/lɔ̃f/	/ʒab/	/mip/			
	/pɛm/	/zat/	/nuʁ/	/vɔʃ/	/duk/			
**6 items sequences**	/dɑ̃ʒ/	/gas/	/miv/	/bɛf/	/nyt/	/ʒyk/		
	/mɔs/	/ʃœʁ/	/ʒov/	/kɛz/	/gun/	/lib/		
**7 items sequences**	/ʁɛp/	/ʒɔʃ/	/lyv/	/zem/	/foz/	/nœʁ/	/ʒib/	
	/vad/	/pyn/	/loz/	/niʒ/	/bɛʁ/	/kɑ̃ʃ/	/fœt/	
**8 items sequences**	/kof/	/ʒam/	/tɛn/	/zyʁ/	/puv/	/fœʁ/	/ʁɑ̃b/	/diʃ/
	/daɲ/	/fɛk/	/pɔs/	/nuv/	/zit/	/ʃyd/	/bɔ̃ʒ/	/ʁœl/

Appendix C

Preferred spellings from the pseudoword spelling task

Table C1

Preferred spellings for each pseudoword from set A per participant

	Set A		
	**Phonological form**	**Possible spellings**	**Preferred spelling per participant**
**Inconsistent Preferred**	/ʒinavə/	ginave	38
		jinave	8
	/ʒebinə/	gébine	43
		jébine	3
	/sedunə/	sédoune	13
		cédoune	33
	/simybə/	simube	26
		cimube	20
	/fapyvə/	fapuve	41
		phapuve	5
	/fedinə/	fédine	41
		phédine	5
	/kityvə/	quituve	23
		kituve	23
	/kemagə/	quémague	22
		kémague	24
**Inconsistent Unpreferred**	/ʒitymə/	gitume	35
		jitume	11
	/ʒevabə/	gévabe	39
		jévabe	7
	/semivə/	sémive	21
		cémive	25
	/sibavə/	sibave	24
		cibave	22
	/fanynə/	fanune	41
		phanune	5
	/fenogə/	fénogue	35
		phénogue	11
	/kidunə/	quidoune	25
		kidoune	21
	/kepydə/	quépude	25
		képude	21

Table C2

Preferred spellings for each pseudoword from set B per participant

	Set B		
	**Phonological form**	**Possible spellings**	**Spelled in the experiment (count)**
**Inconsistent Preferred**	/ʒimunə/	gimoune	31
		jimoune	15
	/ʒedavə/	gédave	38
		jédave	8
	/sitavə/	sitave	17
		citave	29
	/sepidə/	sépide	19
		cépide	27
	/fabogə/	fabogue	41
		phabogue	5
	/fenybə/	fénube	37
		phénube	9
	/kipynə/	quipune	25
		kipune	21
	/kenivə/	quénive	22
		kénive	24
**Inconsistent Unpreferred**	/ʒitogə/	gitogue	34
		jitogue	12
	/ʒenyvə/	génuve	40
		jénuve	6
	/sidynə/	sidune	27
		cidune	19
	/sebavə/	sébave	20
		cébave	26
	/fapunə/	fapoune	40
		phapoune	6
	/febadə/	fébade	39
		phébade	7
	/kimavə/	quimave	28
		kimave	18
	/kepanə/	quépane	19
		képane	27
